# Supplementary material for: Cooperative dynamic polaronic picture of diamond color centers
Source: Nat Commun. 2024 Aug 30;15:7174. doi: 10.1038/s41467-024-51366-x (PMC11364646; doi:10.1038/s41467-024-51366-x)
Supplement: Supplementary file 1 — Supplementary informations [file 41467_2024_51366_MOESM1_ESM.pdf]

Supplementary Information

Cooperative dynamic polaronic picture of diamond color centers

Takuto Ichikawa[1], Junjie Guo[1], Paul Fons[2], Dwi Prananto[3], Toshu An[3], and Muneaki Hase[1]

[1]*Department of Applied Physics, Faculty of Pure and Applied Sciences, University of Tsukuba, 1-1-1 Tennodai, Tsukuba, Ibaraki 305-8573, Japan.*

[2]*Department of Electronics and Electrical Engineering, Faculty of Science and Technology, Keio University, 3-14-1 Hiyoshi, Kohoku-ku, Yokohama, Kanagawa 223-8522, Japan.*

[3]*School of Materials Science, Japan Advanced Institute of Science and Technology, Nomi, Ishikawa 923-1292, Japan.*

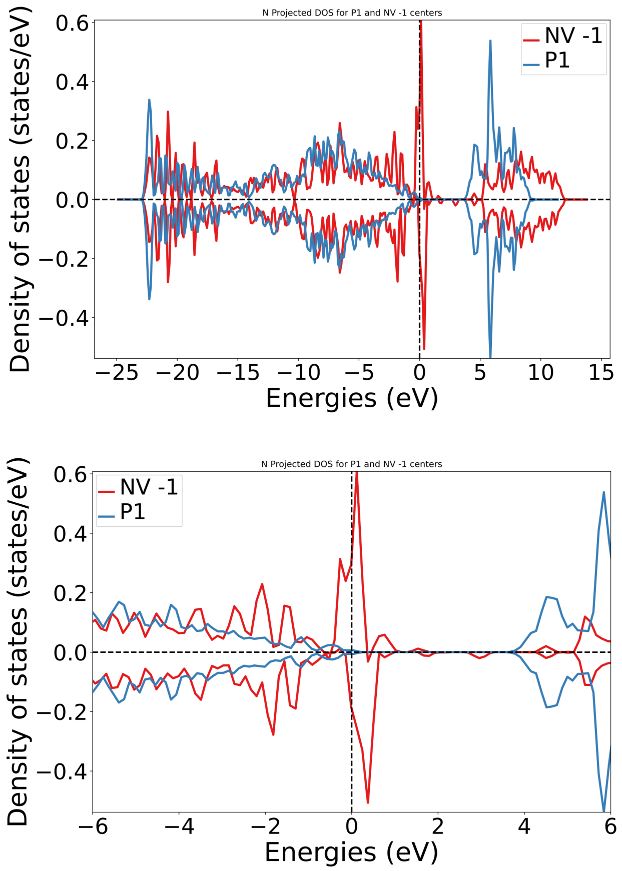

Figure S1: DFT calculations of the density of states (DOS) for NV diamond using VASP 6.4 code with a 64 atoms supercell. The top panel presents a wider energy range, while the bottom panel focuses on the vicinity of the band gap. NV-1 represents the DOS for NV^- center and P1 represents the DOS for the P1 center.

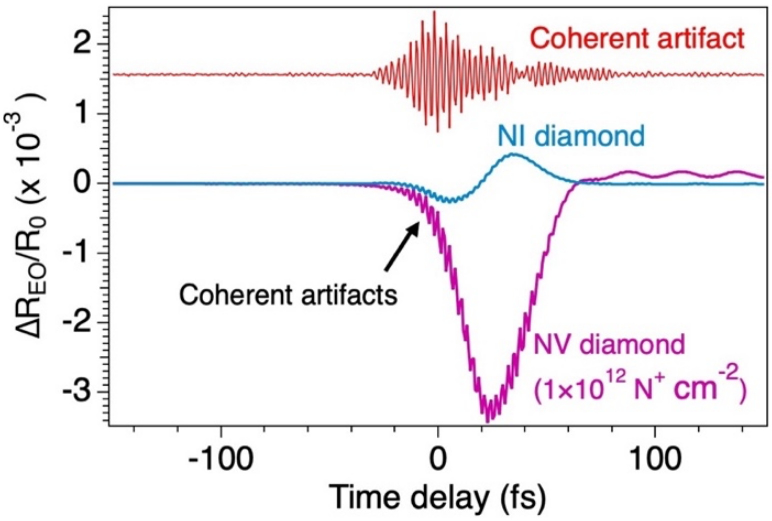

Figure S2: Time-resolved EO response around $\tau = 0$, demonstrating the appearance of coherent artifacts at $\tau = 0$. The top fringe-like coherent artifacts were extracted from the EO response for the NI diamond.

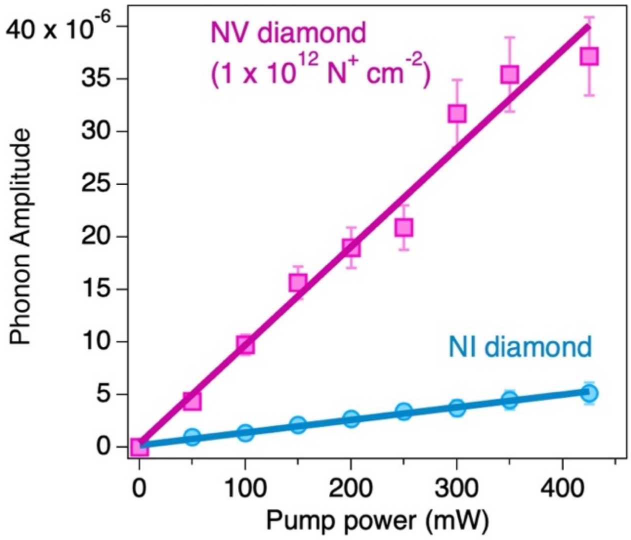

Figure S3: The amplitude of coherent LO phonons as a function of the pump power for the NI and NV ($1x10^{12}$ N^+ cm^{-2}) diamonds. The solid lines are the fit using a linear function. The maximum power in the additional measurements was 430 mW, slightly below that of the main text due to the laser conditions.

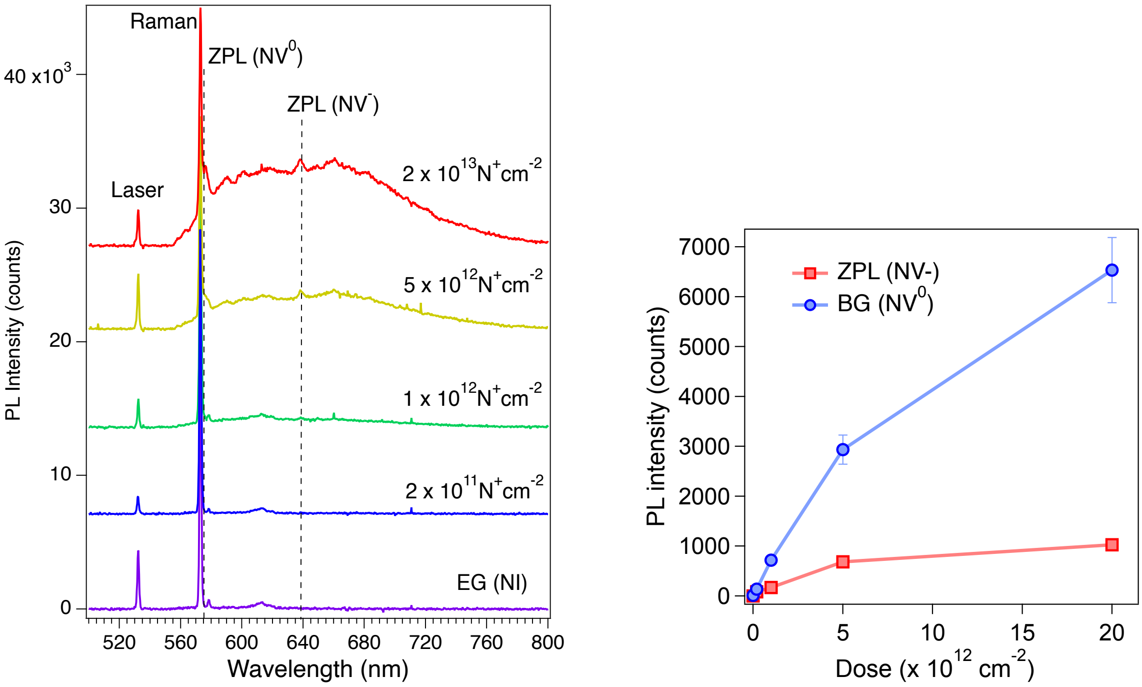

Figure S4: (Left) Photoluminescence spectra obtained for the diamond samples using a 532 nm CW laser and a fiber coupled visible spectrometer. (Right) PL intensity as the function of N^+ dose for zero phonon line (ZPL) at 638 nm of NV^- center and background component at 600 nm reflecting the NV^0 center.

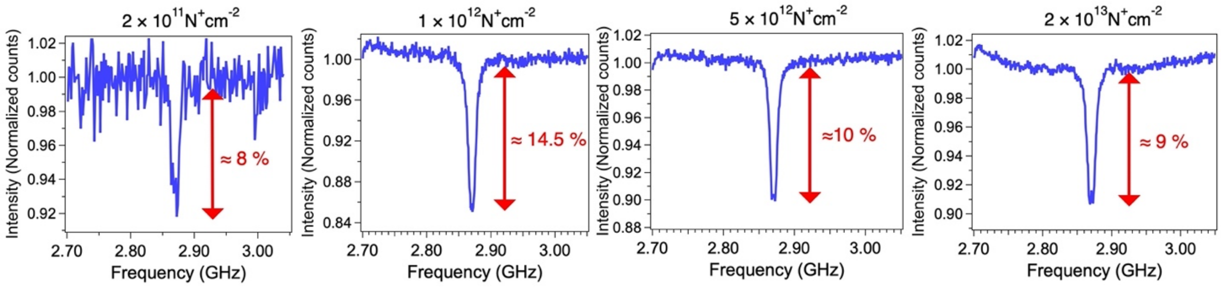

Figure S5: Optically detected magnetic resonance (ODMR) spectra obtained at room temperature for the NV diamond samples using 532 nm cw laser.
